# Pharmacokinetics of Snake Venom

**DOI:** 10.3390/toxins10020073

**Published:** 2018-02-07

**Authors:** Suchaya Sanhajariya, Stephen B. Duffull, Geoffrey K. Isbister

**Affiliations:** 1 Clinical Toxicology Research Group, University of Newcastle, Newcastle 2298, Australia; Suchaya.Sanhajariya@uon.edu.au; 2 School of Pharmacy, University of Otago, Dunedin 9016, New Zealand; stephen.duffull@otago.ac.nz

**Keywords:** snakes, venom, pharmacokinetics, elapid, viper, toxins, This review investigates the current knowledge of snake venom pharmacokinetics. In addition we conducted an exploratory population pharmacokinetic analysis of timed venom concentration data extracted from the literature, which can be used as a prior for the future development of substantial snake venom pharmacokinetic models.

## Abstract

Understanding snake venom pharmacokinetics is essential for developing risk assessment strategies and determining the optimal dose and timing of antivenom required to bind all venom in snakebite patients. This review aims to explore the current knowledge of snake venom pharmacokinetics in animals and humans. Literature searches were conducted using EMBASE (1974–present) and Medline (1946–present). For animals, 12 out of 520 initially identified studies met the inclusion criteria. In general, the disposition of snake venom was described by a two-compartment model consisting of a rapid distribution phase and a slow elimination phase, with half-lives of 5 to 48 min and 0.8 to 28 h, respectively, following rapid intravenous injection of the venoms or toxins. When the venoms or toxins were administered intramuscularly or subcutaneously, an initial absorption phase and slow elimination phase were observed. The bioavailability of venoms or toxins ranged from 4 to 81.5% following intramuscular administration and 60% following subcutaneous administration. The volume of distribution and the clearance varied between snake species. For humans, 24 out of 666 initially identified publications contained sufficient information and timed venom concentrations in the absence of antivenom therapy for data extraction. The data were extracted and modelled in NONMEM. A one-compartment model provided the best fit, with an elimination half-life of 9.71 ± 1.29 h. It is intended that the quantitative information provided in this review will provide a useful basis for future studies that address the pharmacokinetics of snakebite in humans.

## 1. Introduction

In 2009, WHO listed snakebite as a neglected tropical disease, recognising its importance alongside many infectious diseases [1]. This is a particularly important public health issue in tropical and subtropical regions [2,3] mostly affecting those who live in rural areas, including the agricultural workforce in developing countries [3,4,5]. Snake envenomation causes significant morbidity and mortality usually requiring hospitalisation and occasionally causing permanent disabilities and in severe cases death [6]. Delayed access to appropriate medical facilities, lack of antivenom, and limited supportive treatments are considered the main contributors to the high morbidity and mortality [3,4].

Venomous snakes with medical importance are predominantly front-fanged, originating from three families: Atractaspididae, Elapidae, and Viperidae [7]. Snakes from the Viperidae family can be further divided into two subfamilies, Viperinae (true vipers) and Crotalinae (pit vipers). In all families, the venom is produced in a specialised gland and typically delivered to the target organism via modified teeth [8,9]. The major functions of snake venoms are to facilitate prey capture by immobilisation or death, to assist with snake digestion, and to act as a defensive mechanism against predators. Snake venoms comprise a mixture of biologically active proteins and polypeptides (comprising approximately 90–95% of a venom load), along with other non-protein components including carbohydrates, lipids, amines, and inorganic salts (Table 1 and Table 2) [7,10,11]. Proteins and polypeptides can be classified into (1) enzymes [e.g., phospholipase A_2_ (PLA_2_), metalloproteases (SVMP), serine proteases (SVSP), and L-amino acid oxidases (LAAO)], and (2) non-enzymatic substances [e.g., three-finger toxins (3FTx), kunitz peptides (KUN), and disintegrins (DIS)]. The composition of snake venoms varies depending on a variety of factors, including snake family, genus and species, geographical location, typical prey type, and age and size of the snake [12]. For instance, 3FTx and PLA_2_ generally predominate in venoms of elapid snakes, while PLA_2_, SVMP, and SVSP are the most common in vipers, and 3FTx are essentially absent in vipers [7]. Recently, Tasoulis and Isbister (2017) comprehensively reviewed the snake venom proteomes from various compositional studies and classified proteins into five groups based on their abundance and ubiquity [7]. In this work, it was determined that more than 90% of elapid and viper venoms are comprised of 10 protein families. Four of these were considered dominant protein families (PLA_2_, SVMP, SVSP, and 3FTx) and six were secondary protein families (Cysteine-rich secretory proteins (CRiSP), LAAO, KUN, DIS, C-type lectins (CTL), and natriuretic peptides (NP)) [7]. Understanding the variations in snake venom composition is important and will affect studies of snake venoms, their pharmacokinetics, and ultimately the development, production, and treatments of venomous snakebites using antivenoms.

Systemic snake envenomation in humans involves the injected venom being absorbed and entering the systemic circulation, which results in a range of clinical effects depending on the particular snake and the venom components [13]. Snakes from the family Elapidae (cobras, kraits, mambas, Australian venomous terrestrial snakes, coral snakes, and sea snakes) and from the family Viperidae (true vipers and pit vipers) are responsible for most severe envenomation in humans [2,6]. The cocktail of biologically active proteins in venoms gives rise to a wide variety of local and systemic effects (Table 1 and Table 2). The local effects range from local pain and swelling to tissue necrosis [13,14]. Local tissue damage is caused by a range of myotoxic and cytotoxic components such as non-catalytic PLA_2_ myotoxins [15,16], SVMP [15,17], and cytotoxins [15], which can lead to extensive tissue necrosis and potentially require debridement and amputation [5]. The systemic effects are potentially life-threatening and include coagulopathy, neurotoxicity, acute kidney injury, and myotoxicity. Venom-induced consumptive coagulopathy is arguably the most common systemic envenoming syndrome worldwide and is caused by many vipers and Australasian elapids [18]. Neurotoxicity occurs mainly following elapids bite and results from a blockade at the neuromuscular junction, resulting in the paralysis of the facial, bulbar, respiratory, and limb muscles that can lead to respiratory failure and death [2,19].

The measurement of snake venom in blood is rarely available in the clinical setting, but has been undertaken in research studies of snakebite. A number of assay techniques have been developed for the detection of snake venom and venom antigens (toxins) in biological samples, including enzyme-linked immunosorbent assay (ELISA) and radioimmunoassay (RIA). Venom assays have been used in research mainly to identify the snake type involved in envenoming and to evaluate the efficacy of an antivenom in binding the free venom [20]. A venom-specific ELISA was first developed by Theakston and colleagues in 1977 [21] and has good sensitivity and specificity, except in some cases where cross-reactivity may occur from closely related species of snakes [22,23,24]. The limits of detection for a snake venom or toxin are reported between 0.1 mcg/L and 20 mcg/L [25,26,27,28,29,30,31,32,33,34]. The intra- and inter-assay coefficients of variability reported are 1 to 20% and 4 to 20%, respectively [26,27,35]. ELISA, in comparison to RIA, is more practical, easier to perform, more cost-efficient, less time-consuming, and confers no health hazard from handling radioisotopes [21,23,24,36]. The ELISA technique has been used to develop snake venom detection kits, which are used as diagnostic tools for assisting clinicians to make decisions about the appropriate antivenom, but tests swabs from the skin for venom, rather than blood [13,23].

The treatment of snake envenomation principally involves the administration of specific antivenoms for the snake species or type involved, and supportive care. Snake antivenoms are antibodies or antibody fragments which are derived from the plasma of animals (typically horses and sheep) that have been immunised with a snake venom [63]. The intravenously injected antibodies (IgG immunoglobulin) or antibody fragments [F(ab’)_2_ or Fab] bind and neutralise free venom in the patient’s plasma, which aims to reverse or prevent further toxic effects. However, animal-derived antivenoms also carry a risk of hypersensitivity reactions because they are foreign proteins, which can result in cutaneous and multiorgan reactions that are potentially life-threatening with severe anaphylaxis [5,63]. Antivenoms remain expensive, and their supply is often limited in some regions, so more effective dosing and antivenom use may improve their cost-effectiveness.

The dose and timing of antivenom administration is still largely empirical and often based on in vitro neutralisation studies in animals, in which the toxic effects differ compared to humans [64]. In practice, the dose administered is determined by the treating clinicians on the basis of subjective symptoms or clinical or laboratory results. New approaches are required to improve antivenom dosing and determine the optimal dose and timing of antivenom administration. The knowledge of snake venom pharmacokinetics (the study of the time course of venom distribution in biological systems) will provide important information about the time course of the exposure to venom. This will allow for a better determination of a sufficient dose and timing of antivenom administration. In particular, it remains unclear how long post-bite the administration of antivenom remains an effective therapeutic intervention.

This paper aims to bring together the current knowledge of the pharmacokinetics of snake venom by reviewing: (1) laboratory studies performed in animals to investigate the time course of venom concentrations in plasma; and (2) reports describing the time course of venom concentrations in humans.

## 2. Results

### 2.1. Pharmacokinetic Studies in Animals

#### 2.1.1. Literature Search

**Figure 1:**
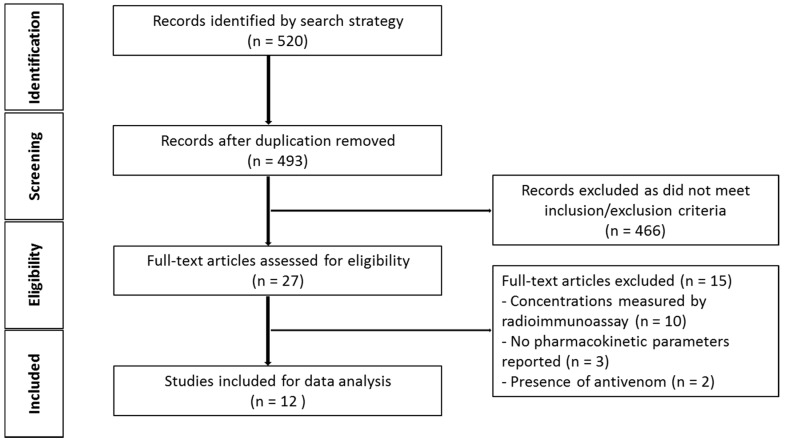
Schematic diagram of article identification (animal studies).

The initial search generated 493 studies for screening, after duplicates were removed. Of these, 481 studies were excluded on the basis of the inclusion and exclusion criteria (see Figure 1). This left 12 studies available for data extraction and to investigate the characteristics and pharmacokinetic parameters of snake venom in animals.

#### 2.1.2. Demographics of Snake Venom Pharmacokinetic Studies in Animals

Of the 12 studies included, eight studies investigated the pharmacokinetics of whole venom, three investigated the pharmacokinetics of isolated snake toxins, and one looked at both venom and purified toxins. Eight of the 12 studies were performed in rabbits. The pharmacokinetic parameters reported in these studies were estimated from the plasma concentration time course following the administration of snake venoms or toxins via the intravenous (IV), intramuscular (IM), or subcutaneous (SC) routes. A summary of the pharmacokinetic parameters reported in these 12 studies are shown in Table 3, Table 4 and Table 5.

#### 2.1.3. Pharmacokinetic Parameters of Snake Venoms and Toxins Following Intravenous Administration

In most studies, the investigators identified a two-compartment output model as the best fit for their data. Overall, regardless of the type of snake, the concentration initially decreased rapidly with a half-life ranging from, for whole venoms, 15 (*Cryptelytrops purpureomaculatus*) to 48 min (*Naja sumatrana* and *Hypnale hypnale*), for toxins, 5 (habutobin of *Trimeresurus flavoviridis*) to 42 min (PLA_2_ of *N. sumatrana*). In the terminal phase, the concentrations decreased more slowly with longer half-lives ranging from, for whole venoms, 12 (*Vipera aspis* and *Bothrops alternatus*) to 28 h (*C. purpureomaculatus*), for toxins, 0.8 (habutobin of *T. flavoviridis*) to 12 h (PLA_2_ of *N. sumatrana*). It is assumed that the initial phase is the distribution and the terminal phase is the elimination, but this cannot be determined from the data presented. Habutobin, a toxin purified from the venom of *T. flavoviridis*, had the shortest distribution and elimination half-lives compared to the rest of the snake venoms and toxins. Also of note, *N. naja atra* cytotoxin had a shorter half-life than the venom and toxins from two other types of cobra studied (*N. sumatrana* and *N. sputatrix*).

Hart et al. (2014) [65] and Paniagua et al. (2012) [66] only reported single values for the half-lives from a one-compartment model. It is interpreted that the disposition phase half-life would be a composite of the distribution and elimination phases reported with the two-compartment models. Tan et al. (2014) [67] reported a three-compartment model fit for their data, with three half-lives (24 min, 48 min, and 19 h, respectively). The half-lives were similar to the two-compartment models.

The reported volume of distribution at steady state (V_ss_) for snake venoms and toxins ranged widely. The V_ss_ of snake venoms ranged from as low as 0.12 L·kg^−1^ in *M. fulvius* venom to 1.2 L·kg^−1^ in *V. aspis* venom. The V_ss_ was notably higher for the thrombin-like enzyme (TLE) from *A. halys ussuriensis Emelianov* venom (1.8 L·kg^−1^).

The systemic clearance (CL) of snake venoms and toxins reported also varied from species to species. The CL of snake venom ranged from 0.007 L·h^−1^·kg^−1^ for *H. hypnale* venom to 0.093 L·h^−1^·kg^−1^ for *M. fulvius* venom, and from 0.048 to as high as 0.324 L·h^−1^·kg^−1^ for snake toxins (PLA_2_ in *N. sumatrana* and TLE from *A. halys ussuriensis Emelianov* venom, respectively). It appears that the reported values of CL of snake toxins are generally larger than those reported for whole snake venoms.

#### 2.1.4. Pharmacokinetic Parameters of Snake Venoms and Toxins Following Intramuscular or Subcutaneous Administration

When snake venom or toxins are administered via the IM or SC routes, the additional influence of absorption rate and bioavailability (F) of venom and toxins needs to be considered. Guo et al., 1993 [27], Audebert et al., 1994 [35], Sim et al., 2013 [74], Yap et al., 2013 [73], Tan et al., 2014 [67], Yap et al., 2014 [70] investigated the pharmacokinetics of snake venom following IM administration, while Zhao et al., 2001 [68] and Paniagua et al., 2012 [66] investigated the pharmacokinetics of snake venom following SC administration. All studies demonstrated evidence of an absorption phase of venom and toxins. Not all venom or toxins were fully absorbed into the circulation following IM administration [F ranging from 4 (*H. hypnale* venom) to 67% (*V. aspis* venom), and from 40 to 82% for *N. sumatrana* toxins] and SC administration [F = 60% for *M. fulvius* venom]. In these studies, the distribution half-life, which is expected to be related to the ongoing absorption that occurs during the initial distribution phase, was not commonly seen. The studies by Zhao et al., 2001 [68] and Guo et al., 1993 [27] are the only two studies that report the half-life of the absorption phase as well as two phases of disposition half-lives. In these two studies, the half-lives following SC administration were reported to be 2.5, 4.8, and 125 h, respectively [68], and those following IM administration were 0.077, 0.37, and 5.9 h, respectively [27].

Guo et al., 1993 [27], Audebert et al., 1994 [35], and Yap et al., 2013 [73] found that the terminal half-life following IM injection was longer than that following IV administration. However, in the studies by Sim et al., 2013 [74], Yap et al., 2013 [73], Yap et al., 2014 [70], and Tan et al., 2014 [67], the terminal half-lives reported following IV and IM administration appeared similar. Both Zhao et al., 2001 [68] and Paniagua et al., 2012 [66] found the terminal half-life to be longer following SC administration than after IV injection. In these cases, it is expected that the prolonged duration of the half-life would be a function of prolonged absorption since the disposition processes should be unaffected by the input processes.

### 2.2. Pharmacokinetic Studies in Humans

#### 2.2.1. Literature Search

**Figure 2:**
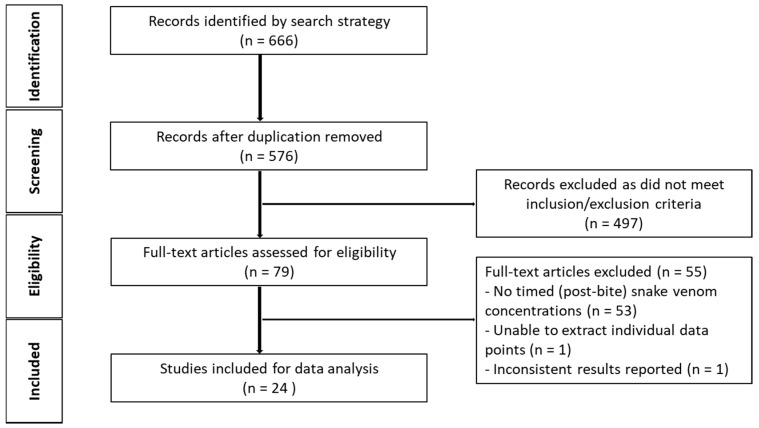
Schematic diagram of article identification (human studies).

The initial search generated 576 studies for screening, after duplicates were removed. A total of 552 studies were further removed on the basis of the inclusion and exclusion criteria (Figure 2), leaving 26 studies for data extraction and ultimately post-hoc (model-based) analysis.

#### 2.2.2. Data Extraction

Timed venom concentration data were extracted from 24 (Table 6) of the 26 studies included from the literature review. One study was not included for further analysis, as the serum antigen concentrations appeared to have increased over time post-bite for all patients over a period of 24 h, which was not consistent with data reported in the other studies. Another study was excluded from data extraction because of the difficulty in distinguishing individual data points from the overlapping concentration-time profiles of the 37 patients, and, hence, we were unable to digitise the data from the figure. Nine studies reported elapid envenomation, and sixteen studies reported viper envenomation.

From the 24 included studies, we were able to retrieve data for 145 individuals. A total of 218 timed concentration data were obtained from the text of the case reports. For studies in which the results were reported graphically, we successfully recreated similar plots by digitising the data points from the figures using the WebPlotDigitizer software.

#### 2.2.3. Data Analysis

**Figure 3:**
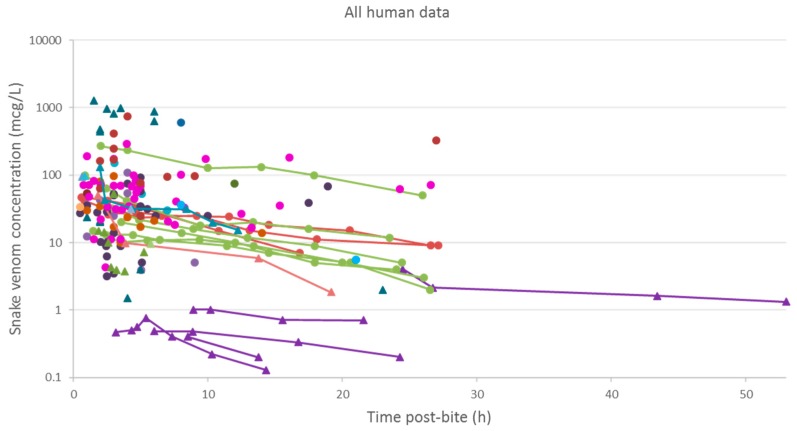
Snake venom concentrations versus time post-bite in 145 patients, extracted from 24 studies. The closed triangles represent data of patients bitten by snakes of the Elapidae family, and the closed circles represents data of patients bitten by snakes of the Viperidae family. The lines are used to join samples from the same patient.

**Figure 4:**
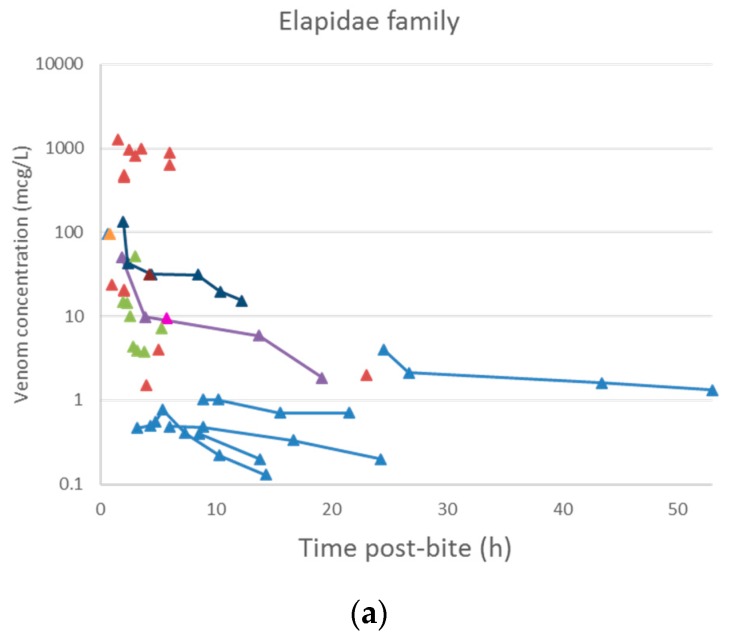

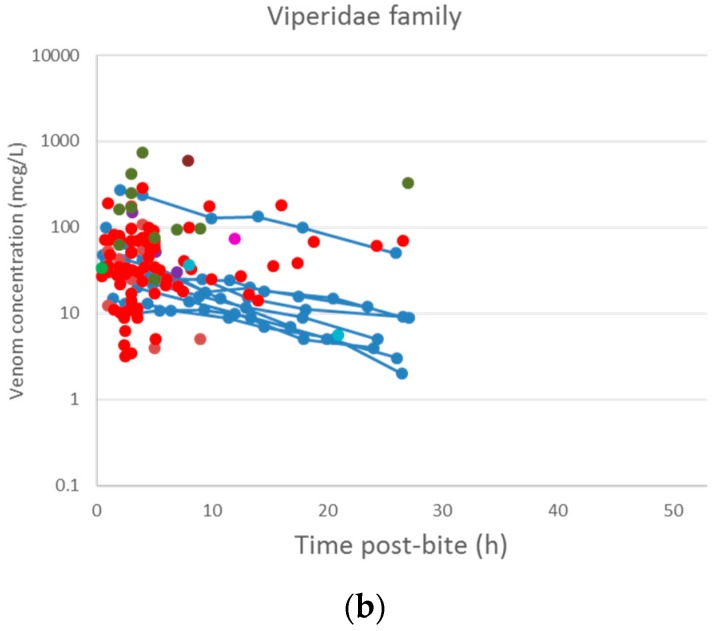
Snake venom concentration versus time data post-bite of patients bitten by snakes of the Elapidae family (**a**) and the Viperidae family (**b**).

A plot of the extracted data is shown in Figure 3 and Figure 4. Most data were collected within the first few hours since snakebite, and most individuals provided only a single timed venom concentration. This is because most studies primarily reported a clinical presentation of snakebite, and only one sample was taken prior to the administration of the antivenom. Only five studies reported serial venom concentrations over time in the absence of antivenom. The dataset obtained was sparse in terms of observations per individual, and there were large magnitudes of difference between the snake venom concentrations. In some cases, snake venom concentrations could still be detected 24 h after the bite. Figure 4 compares the concentration-time profiles of patients bitten by snakes from the Elapidae family (Figure 4a) and the Viperidae family (Figure 4b). It can be seen that the venom concentrations detected in patients bitten by vipers are typically higher than those in patients bitten by elapids.

**Figure 5:**
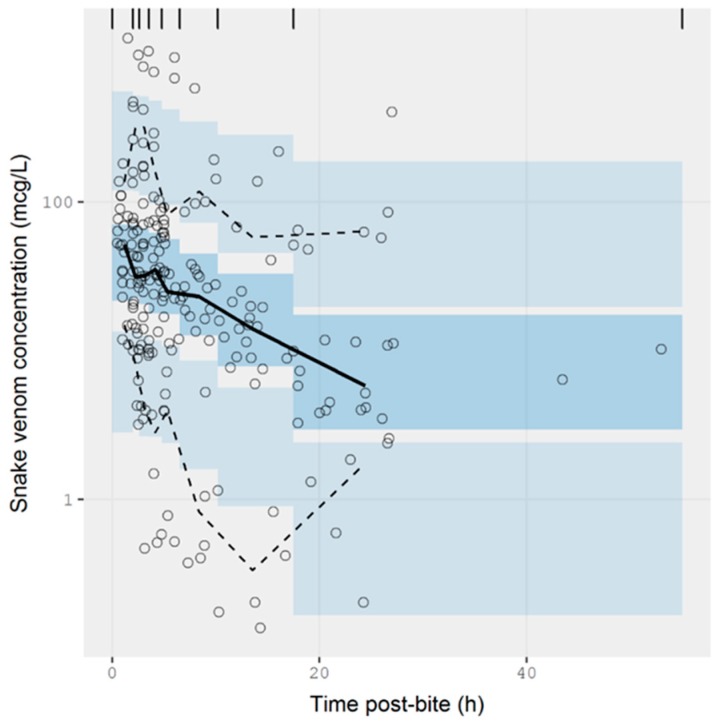
Simulation-based diagnostic plots for snake venom concentration (mcg/L) versus time post-bite (h) for the pharmacokinetic model. The solid line represents the median observed snake venom concentration, and the dashed lines represent the observed 10% and 90% percentile. The shaded areas are the corresponding 95% confidence intervals of the percentiles predicted by the model.

The extracted data were modelled using NONMEM. The concentration-time data were best described by a one-compartmental model with zero-order input and first-order elimination. A prediction-corrected visual predictive check is presented in Figure 5. The observed percentiles are mostly within the 95% confidence interval of simulated percentiles, suggesting that the model provides a good description of the data. The half-life calculated from the estimated values of clearance and volume of distribution was 9.71 ± 1.29 h (see Table A1 of Appendix A for parameter estimates from the model). The model also accounted for the relative exposure (i.e., “dose”) of patients, resulting from bites of snakes from the Elapidae and Viperidae family. It was apparent from these data that vipers inject approximately 75% more venom than elapids, but it should be noted the between-subject variability in the exposure was large, corresponding to 275%, meaning that the variability between bites within a family is at least as significant as the differences between families of snakes. It is possible that some of this variability could be further explained by the snake species, but these data were not available.

## 3. Discussion

### 3.1. Pharmacokinetic Studies in Animals

To date, there is only a handful of experimental studies on the pharmacokinetics of snake venom in the absence of antivenom. Most of these studies were performed in large animals, commonly rabbits, as they allowed for the analysis of multiple samples over a longer period of time, consistent with the duration of envenomation. Since a snake venom is comprised of a cocktail of protein components, it is common that snake venoms are investigated as whole venom rather than as individual toxins. Some investigators took the additional step of isolating the major toxins of interest in order to study their profiles [68,69,70]. For the purpose of this review, we only assessed the pharmacokinetic parameters that arose from studies carried out using ELISA, although there were some other studies performed using RIA methods [94,95,96].

The pharmacokinetic profiles of snake venoms and isolated toxins were investigated in animals following intravenous injection. This avoids issues related to bioavailability and provides profiles that are dependent only on distribution and clearance. Most studies demonstrated that the concentration-time profiles of snake venoms and toxins from both snake families were best described by a two-compartment model. The concentrations reportedly declined rapidly during the initial phase. This suggests that the venoms (or toxins) were rapidly distributed out of the central compartment to peripheral compartments. The initial half-life values reported for the whole venoms and the isolated toxins ranged from 5 min to 48 min, which is significantly faster than the terminal half-lives which could be as long as 28 h. Given the low CL values and the relatively high V_ss_ values of the venoms, we suspect that the terminal phase half-life from the two-compartment model, may be predominantly contributed to by elimination. This does not rule out further distribution components. The volume of distribution of snake venom in animals was relatively large when compared to their blood volume (approximately 0.054–0.070 L·kg^−1^ for rats, 0.057–0.065 L·kg^−1^ for rabbits, and 0.058–0.064 L·kg^−1^ for sheep), which supports the significant distribution of the venom outside of blood. This means that an antivenom will be much less effective (potentially ineffective) in binding the venom once the venom leaves the circulation. Understanding the distribution of a venom between the central compartment and the peripheral compartments and, importantly, the rate at which a venom moves out of the central compartment is essential to determining the effective timing of antivenom administration.

Pharmacokinetic studies in animals were also performed following intramuscular or subcutaneous injection in order to mimic the real-life nature of snakebites. The initial phase of the concentration-time profile was governed by the absorption and simultaneous distribution of the snake venom (and toxins). Some studies observed multiple absorption phases which may be due to the difference in the absorption rate of venom components with a wide range of molecular weights (Table 1 and Table 2). In addition, the lymphatic absorption from the injection site to the blood compartment may be associated with a delayed increase in venom concentration observed in these studies. The absorption of venom appeared to be relatively slow, on the basis of the prolonged elimination half-lives reported, especially following SC administration. This indicates that the absorption is likely to be the rate-limiting step for snake venom disposition, and that the ‘flip-flop’ phenomenon may govern the pharmacokinetics of snake venom. In contrast, some studies reported a similar elimination half-life following IV and IM injection, which indicates that the slow elimination is likely to be the rate-limiting factor of the terminal phase of snake venom time course.

Not all snake venoms are absorbed from the injection site, with their bioavailability being as low as 4% in some cases and as high as 81.5% in other cases. The unabsorbed components of snake venom retained at the injection site may possibly be responsible for local tissue damage. Some confounding estimates of V_ss_ characterized by large values were observed in some studies (Guo et al., 1993 [27], Zhao et al., 2001 [68] and Paniagua et al., 2012 [66]), compared with the V_ss_ following IV injection.

It is important to keep in mind that snake venom is a mixture of large and small molecular weight molecules and that there are limitations in using ELISA to measure such a mixture, because multiple different antibodies are used to detect multiple different antigens. Therefore, the venom concentrations measured and the pharmacokinetic parameters obtained in these studies are an averaged representation of the venom profile and may not capture the true characteristics or variability of the various snake toxins in the biological system. High-performance liquid chromatography with mass spectrometry would provide a more accurate measure of each toxin type, but such an approach to venom measurement has not been undertaken to date.

### 3.2. Pharmacokinetic Studies in Humans

In this meta-analysis, we were able to model literature-derived data to explore the pharmacokinetic parameters of snake venom for the first time. Timed venom concentration data from the literature were obtained from pre-antivenom samples described mainly in studies investigating patients who were intended to be administered an antivenom. The data extracted from the literature and modelled were best described by a one-compartment model. It is likely that venom may well display multicompartment pharmacokinetics in humans, but the available data that could be extracted were too sparse to support more than a one-compartment model. In this work, we also identified a bite-dependent exposure related to snakes of different families, indicating that the Viperidae family injects approximately a 75% higher dose. In the model, we noted a large between-subject variability in the relative dose injected (indicated as the between-subject variability in F1 from Table A1 of Appendix A). The variability between subjects in the remaining parameters, e.g., CL and V, was not wholly different from that of therapeutic drugs for which the normal variability in CL is approximately 50% in the case of small drug molecules [97]. In the model, the elimination half-life was calculated to be approximately 10 h. Of note, Audebert et al., 1994 also reported similar findings, showing a mono-exponential decline of snake venom concentration, with elimination half-lives ranging from 5 h to 12 h [75]. However, our results are in contrast to those of most animal studies in which two-compartment model fits are reported and hence the terminal half-life is often prolonged more than what observed here. Larger and richer venom concentration datasets in human envenoming are required to support multicompartment models and explore between-subject and between-snake variability, as well as other potential pharmacokinetic covariates.

When comparing our results to an allometrically scaled human half-life from animal data following IV administration of venom, a significant interspecies variability is observed. The allometric scaling of half-life is calculated as:

(1) $$t1/2\;\left(human\right)=t1/2\left(animal\right)\times {\left(\frac{WT_{human}}{WT_{animal}}\right)}^{0.25}$$

where *WT* is the weight of the human (assumed 70 kg) or animal (assumed 0.31 kg for rats and 2.3 kg for rabbits) examined. Considering a terminal half-life of 5.3 h in rats (derived as the arithmetic average from the data in Table 3) and 16 h in rabbits (derived as the arithmetic average from the data in Table 3), the allometrically scaled half-life in humans was estimated to be 21 h and 39 h. The terminal phase half-lives calculated from rat and rabbit data were significantly longer compared to those in our study. The discrepancy in these results may be due to the sparse human data only supporting a one-compartment model compared to the two-compartment models for the rats and rabbits, considering that the half-life from the one-compartment model will be shorter than the terminal phase half-life from the two-compartment model. It is possible, therefore, that the terminal half-life in humans could be longer than one day.

Further analysis needs to be performed to account for these differences and to identify outstanding covariates that may inform the terminal phase of exposure.

## 4. Conclusions

While there is a limited number of studies that investigated the pharmacokinetics of snake venom in animals and reported venom concentrations in humans, this review yields important quantitative information which can be used as a basis for the future development of snake venom pharmacokinetic models. A meta-analysis of snake venom pharmacokinetics in humans has led to the development of a preliminary model, which was able to describe literature-derived data. Further exploration of the relationship between parameters and covariates is necessary to examine the factors that may influence the pharmacokinetic profiles of snake venom and construct a substantial model from data in a larger series of snake envenomation cases. It is surprising that a relatively meaningful analysis was possible, despite the limitations of ELISA, the fact that venom is a mixture of small and large molecules, and the differences in snakes. The development of improved methods to measure venom is required, as well as studies focusing on single snake species or on similar types of snakes.

## 5. Methods

### 5.1. Pharmacokinetic Studies in Animals

We reviewed pharmacokinetic studies of the administration of snake venom to animals and summarised relevant data.

#### 5.1.1. Literature Search Strategy

EMBASE (1974–present) and Medline (1946–present) were used to identify relevant articles. Keywords and text terms included “exp envenomation”, “exp snakebite”, “exp snake venom”, “exp pharmacokinetics”, “exp toxicokinetics”. The search was limited to the English language and animals.

#### 5.1.2. Inclusion and Exclusion Criteria

The publications were only included if they identified the venom or toxins administered, provided timed venom and toxin concentrations (in the absence of antivenom) or a compartmental analysis of their data measured by ELISA, and conducted a pharmacokinetic analysis. Studies that measured venom concentration using RIA were not included because of the possibility of an inaccurate determination of the pharmacokinetic parameters as a result of the metabolism or of the degradation of the radio-labelled proteins [98]. Review articles, commentaries, editorial papers, and conference abstracts were excluded.

#### 5.1.3. Data Extraction and Creation of a Summary Table

The studies meeting all the inclusion criteria were grouped according to animal species. The pharmacokinetic parameters extracted from these publications included: half-life, bioavailability, clearance, and volume of distribution. A summary table was created showing the demographics of the animal studies, snake identification or venom or toxin type, type and number of animals, route of administration, dose administered, and pharmacokinetic parameters. When different units of doses or parameters were reported in the studies, they were converted to mcg·kg^−1^ for the dose administered, hours for the half-lives, L·kg^−1^ for the volume of distribution at steady state (V_ss_), and L·h^−1^·kg^−1^ for the systemic clearance (CL).

### 5.2. Pharmacokinetic Studies in Humans

We reviewed all pharmacokinetic studies of snake venom in humans and extracted relevant data for further analysis.

#### 5.2.1. Literature Search Strategy

Both EMBASE (1974–present) and Medline (1946–present) databases were used for the search. Keywords and text words used for the search were “exp envenomation”, “exp snakebite”, “exp snake venom”, “exp venom antiserum”, “exp snake venom antiserum”, “exp antivenin”, “exp enzymelinked immunosorbent assay”, “exp enzyme immunoassay”, “exp immunoenzyme technique”.

The publications were only included for data extraction if they reported the identity of the snake, timed (post-bite) venom concentrations (in the absence of antivenom), and used ELISA to measure the snake venom concentrations. The review was limited to English language and humans. Review articles, commentaries, editorial papers, and conference abstracts were excluded.

#### 5.2.2. Data Extraction and Synthesis

The snake venom concentration-time data post-envenomation were extracted by (1) obtaining the reported concentrations from the written text, or (2) digitising relevant data points from the concentration-time graphs published in the paper. The graphical data were digitised using WebPlotDigitizer v3.12 software (available at: http://arohatgi.info/WebPlotDigitizer/). All data points considered to have occurred after the administration of an antivenom were excluded.

#### 5.2.3. Data Analysis

The extracted data were transferred to a Microsoft Excel spreadsheet and replotted as a concentration-time profile of different snake venoms from different studies. Since the data included repeated measures on many individuals, the data were modelled within a nonlinear mixed-effects modelling framework using NONMEM 7.2 (ICON Development Solutions, Ellicott City, MD, USA), and standard pharmacokinetic models were considered. The best model was determined using the likelihood ratio test, with degrees of freedom equal to the number of parameters different in successive models. The final model was evaluated using a prediction-corrected visual predictive check.

## Figures and Tables

**Table 1:** List of common venom components (enzymatic) found in snakes of the Elapidae and Viperidae families, their size and activities.

Enzymatic Components	Approximate Molecular Mass (kDa)	Mechanism of Action	Examples of Biological Effects	Snake Families
Phospholipase A_2_ (PLA_2_)	12–14 [37]	Hydrolyses the ester bond at sn-2 position of phospholipids producing free fatty acids and lysophospholipid. Toxic effects can result from this enzymatic action or may be the results of non-enzymatic activity [38].	Myotoxicity, oedema formation, anticoagulant effects, hypotension, presynaptic neurotoxicity [16,39]	Elapidae (type I PLA_2_); Viperidae (type II PLA_2_) [7]
Snake-venom metalloproteases (SVMP)	Classified into 3 groups based on domain organisation [40]; P-I: 20–30; P-II: 30–60; P-III: 60–100	Proteolytic activities leading to degradation of protein structures e.g., basement membranes of blood vessel and extracellular matrix components [17,41]. Disintegrin-like domain of SVMP may also contribute to the haemorrhagic effects [17].	Induce local and systemic bleeding and disrupt haemostasis through its pro-/anticoagulation properties. Extravasation of blood, inflammation and tissue necrosis [17,41]	Major protein family in viper venoms, but less abundant in elapid venom [7]
Serine proteases (SVSP) e.g., thrombin-like enzymes	26–67 [42]	Hydrolyse peptide bonds mainly in pro-enzymes in the coagulation cascade, causing procoagulant, fibrinolytic and/or fibrinogenolytic activities. Some SVSPs have kallikrein-like activity leading to release of bradykinin [42,43].	Disruption of haemostasis and hypotension [42]	Almost all Viperidae, uncommon in Elapidae except Australian snakes [7]
l-amino acid oxidases (LAAO)	110–150 when measured by gel-filtration method under non-denaturing conditions; 50–70 when measured by SDS/PAGE method under reducing and non-reducing conditions [44]	Catalyse stereospecific oxidative deamination of l-amino acid, resulting in production of α-keto acid, ammonia and hydrogen peroxide [45].	Effects on platelet aggregation, inducing cell apoptosis, and antimicrobial activities [46]	Both Elapidae and Viperidae. Most common in Crotalinae [7].
5′-Nucleotidases	53–82 [47]	Hydrolyse phosphate monoester linked to 5′-position of DNA and RNA [48].	Platelet aggregation inhibition [49,50]	Both Elapidae and Viperidae [48]
Acetylcholinesterases	55–60 [47]	Hydrolyse acetylcholine to choline and acetate group [51].	Termination of neurotransmission by acetylcholine [51,52]	Elapidae except *Dendroaspis* genus [52]
Hyaluronidases	33–110 [45]	Hydrolyse hyaluronan into oligosaccharides and *N*-acetylglucosamine [45].	“Spreading factor” alters the structural, rheological, and chemical properties of the extracellular matrix [45]	Both Elapidae and Viperidae [48]

Abbreviations: PLA_2_, phospholipase A_2_; SVMP, snake venom metalloproteases; SVSP, snake-venom serine proteases; LAAO, l-amino acid oxidases.

**Table 2:** List of common venom components (non-enzymatic) found in snakes of the Elapidae and Viperidae families, their size and activities.

Non-Enzymatic Components	Approximate Molecular Mass (kDa)	Mechanism of Action	Examples of Biological Effects	Snake Families
Three-finger toxins (3FTx) e.g., α-neurotoxins	6–9 [47]	Inhibit postsynaptic nicotinic acetylcholine receptors in neuromuscular junction and interfere with neuromuscular transmission [53,54]. Other activities include cardiotoxins, L-type calcium channel blockage, inhibition of platelet aggregation [53].	Postsynaptic neurotoxicity	Elapidae and very rare in Viperidae [7]
Kunitz peptides (KUN)	7 [55,56]	Inhibit serine protease (e.g., trypsin and plasmin) activities, interfering with blood coagulation and fibrinolysis [55,56]. Other activities include ion channel blockade and inflammation [56].	Disruption of haemostasis	Elapidae and Viperinae (absent in Crotalinae) [7]
Cysteine-rich secretory proteins (CRiSP)	20–30 [57]	L-type calcium and cyclic nucleotide-gated (CNG) channel blockade [57].	Inhibit smooth muscle contraction [57]	More common and abundant in Viperidae [7]
C-type lectins (CTL)	Composed of two subunits [58]; α (A chain): 14–15; β (B chain): 13–14	Bind to, inhibit, or activate specific platelet membrane receptors or blood coagulation factors [59].	Anticoagulation, promote or inhibit platelet aggregation [59]	More abundant in Viperidae [7]
Disintegrins (DIS)	5–10 [60]	Bind to glycoprotein IIb/IIIa (α_IIb_β_3_ integrin) expressed on activated platelet to prevent interaction with fibrinogen [60].	Inhibit platelet aggregation [60]	Viperidae, absent in Elapidae [7]
Natriuretic peptides (NP)	3.5–4 [61]	Interaction between NPs and guanylyl cyclase receptors leads to an increase in cGMP levels and subsequent signalling cascade [62]. NPs can also affect renin-angiotensin system by inhibiting angiotensin-converting enzyme [61].	Vasodilation, diuresis, and natriuresis leading to hypotension, and promote sodium and water excretion [62]	Both Elapidae (atrial-type and brain-type) and Viperidae (C-type) [5]. More common and abundant in Viperidae than Elapidae [7]

Abbreviations: 3FTx, three-finger toxins; KUN, Kunitz peptides; CRiSP, Cysteine-rich secretory proteins; CTL, C-type lectins; DIS, disintegrins; NP, natriuretic peptides.

**Table 3:** Pharmacokinetic parameters of snake venom and toxins following intravenous (IV) injection expressed as mean (±SD).

	Snake Species	Animal Model	No.	Dose (mcg·kg^−1^)	t_1/2α_ (h)	t_1/2β_ (h)	V_ss_ (L·kg^−1^)	CL (L·h^−1^·kg^−1^)	Ref
***Isolated Toxin***									
**Rats**									
	*Agkistrodon halys ussuriensis Emelianov*	Sprague-Dawley rats of either sex (180–200 g)	5	50 (thrombin-like enzyme)	0.3 (±0.12 *)	3.9 (±1.63 *)	1.8 (±1.03 *)	0.324 (±0.067 *)	[68]
**Rabbits**									
	*Naja naja atra*	New Zealand rabbits of either sex (1.82 ± 0.09 kg)	6	200 (cytotoxin)	0.097 (±0.01)	3.5 (±0.2)	1.7 ^a^ (±0.3)	0.185 ^b^	[27]
	*Trimeresurus flavoviridis*	Japanese white rabbits (3.2–4.4 kg)	5	50 (habutobin)	0.074 (±0.021 **)	0.84 (±0.13 **)	0.031 ^b,c^, 0.021 ^b,d^	0.061 ^b^	[69]
	*Naja sumatrana*	New Zealand white rabbits (approx. 2 kg)	3	50 (PLA_2_)	0.7 (±0.03)	11.7 (±0.8)	0.25 ^b,c^, 0.45 ^b,d^	0.048 ^b^	[70]
				50 (neurotoxin)	0.5 (±0.1)	8.8 (±0.9)	0.45 ^b,c^, 0.5 ^b,d^	0.082 ^b^	
				50 (cardiotoxin)	0.6 (±0.1)	8.6 (±0.1)	0.5 ^b,c^, 0.55 ^b,d^	0.087 ^b^	
				100 (cardiotoxin in whole venom)	0.5 (±0.01)	11.0 (±0.2)	0.4 ^b,c^, 0.5 ^b,d^	0.060 ^b^	
***Whole Venom***									
**Rats**									
	*Bothrops alternatus*	Male Wistar rats (200–250 g)	6	800	0.38 (±0.03)	12.1 (±6.4)	0.50 (±0.12)	0.033 (±0.011)	[71]
	*Pseudechis australis*	Male Sprague-Dawley rats (320–420 g)	8	100	-	0.27 ***	-	-	[65]
**Rabbits**									
	*Vipera aspis*	Charles de Bouscat HY rabbits (2.5–3 kg)	5	250	0.71 (±0.2 *)	12 (±2.24 *)	1.2 (±0.089 *)	0.084 (±0.013 *)	[35]
	*Vipera aspis*	New Zealand rabbits (2.75–3 kg)	5	250	0.53 (±0.31 *)	14.2 (±2.68 *)	0.7 (±0.11 *)	0.040 (±0.002 *)	[72]
	*Naja sputatrix*	New Zealand rabbits (2 kg)	3	90	0.5 (±0.3)	15.4 (±2.5)	0.8 ^b^	0.034 ^b^	[73]
	*Cryptelytrops purpureomaculatus*	Male New Zealand rabbits (1.7–2.1 kg)	3	200	0.25 (±0.01)	27.7 (±0.0)	0.39 ^c^ (±0.01), 1.80 ^d^ (±0.11)	0.055 (±0.003)	[74]
	*Naja sumatrana*	New Zealand white rabbits (approx. 2 kg)	3	100	0.8 (±0.3)	13.6 (±1.1)	0.5 ^b,c^, 0.4 ^b,d^	0.046 ^b^	[70]
	*Hypnale hypnale*	New Zealand white rabbits (1.95 ± 0.05 kg)	3	10	0.8 (±0.17 *)	19.3 (±3.29 *)	0.13 ^b^	0.007 (±0.001 *)	[67]
**Sheep**									
	*Micrurus fulvius*	Sheep (36–60 kg)	4	1000 mcg	-	0.42 (±0.11 *)	0.12 ^b^	0.093 ^b^	[66]

* SD calculated from standard errors reported in the original papers using the following equation: SD=SE × √n; ** Assumed to be reported as SD; *** SD not reported; ^a^ = Apparent volume of distribution; ^b^ = Unit by weight (·kg^−1^) calculated on the basis of the mean of the reported animal weight; ^c^ = Apparent volume of central compartment by weight (V_c_); ^d^ = Apparent volume of peripheral compartment by weight (V_p_); Abbreviations: t_1/2α_, half-life of the distribution phase; t_1/2β_, half-life of the elimination phase; V_ss_, apparent volume of distribution at steady state; CL, systemic clearance.

**Table 4:** Pharmacokinetic parameters of snake venom and toxins following intramuscular (IM) injection expressed as mean (±SD).

	Snake Species	Animal Model	No.	Dose (mcg·kg^−1^)	t_1/2ka_ (h)	F (%)	t_1/2α_ (h)	t_1/2β_ (h)	V_ss_ (L·kg^−1^)	CL (L·h^−1^·kg^−1^)	Ref
***Isolated Toxin***											
**Rats**											
	*Naja naja atra*	New Zealand rabbits of either sex (1.82 ± 0.09 kg)	6	500 (cytotoxin)	0.077 (±0.018)	-	0.37 (±0.12)	5.9 (±0.9)	9 ^a^ (±4)	0.56 ^b^	[27]
**Rabbits**											
	*Naja sumatrana*	New Zealand white rabbits (approx. 2 kg)	3	100 (PLA_2_)	-	68.6 (±0.8)	-	10.18 (±1.18)	-	0.048 ^b^	[70]
				70 (neurotoxin)	-	81.5 (±0.6)	-	8.6 (±0.5)	-	0.082 ^b^	
				150 (cardiotoxin)	-	45.6 (±0.1)	-	8.2 (±0.1)	-	0.087 ^b^	
				500 (cardiotoxin in whole venom)	-	39.5 (±1.1)	-	11.6 (±0.9)	-	0.061 ^b^	
***Whole Venom***											
**Rabbits**											
	*Vipera aspis*	Charles de Bouscat HY rabbits (2.5–3 kg)	5	300	-	63 (±17.89 *)	-	32 (±8.94 *)	-	-	[35]
				500	-	67 (±11.18 *)	-	36 (±8.94 *)	-	-	
				700	-	63 (±38.01 *)	-	29 (±4.47 *)	-	-	
	*Naja sputatrix*	New Zealand rabbits (2 kg)	3	500	-	41.7 **	-	18.9 (±4.6)	-	0.034 ^b^	[73]
	*Cryptelytrops purpureomaculatus*	Male New Zealand rabbits (1.7–2.1 kg)	3	500	-	41.6 (±3.0)	-	27 (±0.6)	-	0.055 (±0.004)	[74]
	*Naja sumatrana*	New Zealand white rabbits (approx. 2 kg)	3	500	-	41.9 (±0.2)	-	12.5 (±0.9)	-	0.047 ^b^	[70]
	*Hypnale hypnale*	New Zealand white rabbits (2.03 ± 0.06 kg)	3	1000	-	4 **	-	19.3 (±1.21 *)	-	0.007 (±0.003 *)	[67]

* SD calculated from standard errors reported in the original papers using the following equation: SD=SE × √n; ** Assumed to be reported as SD; ^a^ = Apparent volume of distribution; ^b^ = Unit by weight (·kg^−1^) calculated on the basis of the mean of the reported animal weight; Abbreviations: t_1/2ka_, half-life of the absorption phase; F, bioavailability; t_1/2α_, half-life of the distribution phase; t_1/2β_, half-life of the elimination phase; V_ss_, apparent volume of distribution at steady state; CL, systemic clearance.

**Table 5:** Pharmacokinetic parameters of snake venom and toxins following subcutaneous (SC) injection expressed as mean (±SD).

	Snake Species	Animal Model	No.	Dose (mcg·kg^−1^)	t_1/2ka_ (h)	F (%)	t_1/2α_ (h)	t_1/2β_ (h)	V_ss_ (L·kg^−1^)	CL (L·h^−1^·kg^−1^)	Ref
***Isolated Toxin***											
**Rats**											
	*Agkistrodon halys ussuriensis Emelianov*	Sprague-Dawley rats of either sex (180–200 g)	6	750 (thrombin-like enzyme)	2.5 (±0.73 *)	-	4.8 (±4.16 *)	125 (±181.26 *)	19 (±49 *)	0.294 (±0.103 *)	[68]
***Whole Venom***											
**Rabbits**											
	*Micrurus fulvius*	Sheep (36–60 kg)	4	5000 mcg	-	60 (±10 *)	-	4.35 (±1.83 *)	0.56 ^b^	0.084 ^b^	[66]

* SD calculated from standard errors reported in the original papers using the following equation: SD=SE × √n; ^b^ = Unit by weight (·kg^−1^) calculated on the basis of the mean of the reported animal weight; Abbreviations: t_1/2ka_, half-life of the absorption phase; F, bioavailability; t_1/2α_, half-life of the distribution phase; t_1/2β_, half-life of the elimination phase; V_ss_, apparent volume of distribution at steady state; CL, systemic clearance.

**Table 6:** List of publications obtained from literature search and the demographics of snakebite cases.

Snake Species	Country	Number of Patients with Timed Concentration Post-Bite for Data Retrieval	Ref
*Pseudonaja spp.*	Australia	5	[25]
*Vipera aspis*, *Vipera berus*, *and Vipera ammodytes*	France	3	[26]
*Vipera aspis and Vipera berus*	France	6	[75]
*Crotalus durissus terrificus*	Brazil	9	[76]
*Vipera ammodytes*	Slovenia	3	[77]
*Bothrops jararaca*	Brazil	1	[78]
*Bothrops jararaca*	Brazil	1	[79]
*Crotalus durissus terrificus*	Brazil	1	[80]
*Bothrops lanceolatus*	Martinique	1	[81]
*Daboia russelli siamensis*	Thailand	24	[28]
*Naja atra*	Taiwan	14	[82]
*Daboia russelli siamensis*	Taiwan	10	[83]
*Pseudonaja spp.*	Australia	1	[84]
*Acanthophis spp.*	Australia	1	[85]
*Denisonia maculata*	Australia	1	[86]
*Vipera russelli*	Myanmar	38	[29]
*Pseudechis porphyriacus*	Australia	1	[87]
*Bitis gabonica*	UK	1	[88]
*Vipera russelli pulchella*	Sri Lanka	1	[89]
*Crotalus durissus*	Brazil	11	[30]
*Cerastes cerastes mutila*	Switzerland & UK	2	[90]
*Bungarus caeruleus*	Sri Lanka	8	[91]
*Ophiophagus Hannah*	Myanmar	1	[92]
*Micropechis ikaheka*	Papua New Guinea	1	[93]

## Appendix A

**Table A1:** Parameter estimates from the pharmacokinetics models.

Parameters	Parameter Estimates (RSE%)	
	Base Model	Covariate Model
CL (L/h)	15.2 (11%)	13.3 (14%)
V (L)	215 (9%)	184 (10%)
D1 (h)	1 FIX	1 FIX
F1 (Viperidae)	1 FIX	1 FIX
F1 (Elapidae)	1 FIX	0.569 (43%)
**Between subject variability**		
CL (CV %)	52.6% (30%)	43.7% (52%)
V (CV %)	15.5% (237%)	29.8% (101%)
D1 (CV %)	44.1% (21%)	44.1% (17%)
F1 (CV %)	286.5% (7%)	275.4% (7%)
**Residual error**		
Proportional error	0.047 (25%)	0.047 (25%)

Abbreviations: CL, clearance; V, apparent volume of distribution; D1, duration for central compartment; F1, bioavailability for central compartment; RSE, relative standard error; CV%, percentage coefficient of variation.
